# The role of the hepatitis B virus genome and its integration in the hepatocellular carcinoma

**DOI:** 10.3389/fmicb.2024.1469016

**Published:** 2024-09-06

**Authors:** Weiyang Li, Suhao Wang, Yani Jin, Xiao Mu, Zhenzhen Guo, Sen Qiao, Shulong Jiang, Qingbin Liu, Xiaofang Cui

**Affiliations:** ^1^Jining Medical University, Jining, China; ^2^School of Biological Science, Jining Medical University, Rizhao, China; ^3^Jining First People's Hospital, Shandong First Medical University, Jining, China; ^4^Clinical Medical Laboratory Center, Jining First People's Hospital, Shandong First Medical University, Jining, China

**Keywords:** hepatitis B virus integration, liver cancer, genomic instability, hepatitis B virus infection, hepatitis B virus

## Abstract

The integration of Hepatitis B Virus (HBV) is now known to be closely associated with the occurrence of liver cancer and can impact the functionality of liver cells through multiple dimensions. However, despite the detailed understanding of the characteristics of HBV integration and the mechanisms involved, the subsequent effects on cellular function are still poorly understood in current research. This study first systematically discusses the relationship between HBV integration and the occurrence of liver cancer, and then analyzes the status of the viral genome produced by HBV replication, highlighting the close relationship and structure between double-stranded linear (DSL)-HBV DNA and the occurrence of viral integration. The integration of DSL-HBV DNA leads to a certain preference for HBV integration itself. Additionally, exploration of HBV integration hotspots reveals obvious hotspot areas of HBV integration on the human genome. Virus integration in these hotspot areas is often associated with the occurrence and development of liver cancer, and it has been determined that HBV integration can promote the occurrence of cancer by inducing genome instability and other aspects. Furthermore, a comprehensive study of viral integration explored the mechanisms of viral integration and the internal integration mode, discovering that HBV integration may form extrachromosomal DNA (ecDNA), which exists outside the chromosome and can integrate into the chromosome under certain conditions. The prospect of HBV integration as a biomarker was also probed, with the expectation that combining HBV integration research with CRISPR technology will vigorously promote the progress of HBV integration research in the future. In summary, exploring the characteristics and mechanisms in HBV integration holds significant importance for an in-depth comprehension of viral integration.

## 1 Introduction

Hepatocellular carcinoma (HCC) is a type of liver cancer originating from liver cells and is one of the most common malignant tumors. The mortality rate of liver cancer ranks third among cancer deaths worldwide (Sung et al., 2021), while it ranks fifth in cancer incidence and second in cancer mortality, in China (Huang et al., 2022). Although the pathogenesis of HCC is still unclear, epidemiological studies have confirmed that HBV is a leading cause of HCC among many risk factors. A higher HBV infection rate may lead to a high frequency of HCC occurrence (Hong et al., 2017), and HBV carriers have a higher risk (5–15 times) of developing cancer than non-carriers (Wang et al., 2021; Yano et al., 2006; El-Serag and Rudolph, 2007). HBV infection has been associated with the development of liver cancer, as it plays a crucial role in the carcinogenesis, invasion, and metastasis of liver cells (Liu et al., 2020; Zha et al., 2018).

## 2 HBV structure and genome

Hepatitis B virus (HBV) belongs to the Hepadnaviridae family. The virus particles, also called dane particles, are spherical in shape, with 42 nm of diameter. The outer capsid is like a typical double-layer viral envelope. The surface antigen (HBsAg) of HBV is embedded in the lipid bilayer of the envelope, while its core structure has relatively high electron density and is 20-hedron stereo symmetric with approximately 27 nm diameter. The inner shell of the virus has an antigenic protein called the core antigen (HbcAg) of HBV (D'Mello et al., 1997).

HBV has been divided into eight genotypes, and each genotype has different geographical distribution characteristics. In China, the common genotypes are B and C. The HBV has an enveloped structure containing a 3.2 kb genome. The circular double-stranded DNA (dsDNA) genome is enclosed in a virally encoded capsid. The genome has high information density and widely overlapping open reading frames (ORFs). The negative strand of the HBV genome contains 4 open reading frames (ORFs), which are P gene, S gene (divided into pre-S2 region, pre-S1 region, S region), the X gene and the C gene (divided into the pre-C region and C region). The ORFs encode seven different proteins, including structural protein, HBsAg and HBV core antigen (HBcAg), preCore, HBV e antigen (HBeAg), HBV polymerase and HBV X protein (HBx) (Tian and Jia, 2016).

## 3 Replication of HBV genome

The first step in the replication of HBV is to invade the human body and adhere to the liver cell membrane using its outer membrane (surface antigen, HBsAg). However, for adhesion process, HBsAg must first recognize the liver cell membrane (Takahashi, 2004). For an efficient infection process, viruses usually employ various receptors or coreceptors on the cell membrane to mediate their infection. In 2012, Wenhui Li discovered and reported sodium taurocholate cotransporter (NTCP) as a functional receptor for HBV infection, which effectively resolved the unclear process of HBV invasion into liver cells. This discovery greatly stimulated the research progress of the mechanism of HBV infection (Toniutto et al., 2024). At present, it is not clear whether any other receptors or coreceptors are involved in the process of HBV infection. In recent years, researchers have taken advantage of the heterogeneity of HBV infection among cells and performed single-cell sequencing and susceptibility analysis on the liver biopsy samples of children not on antiviral therapy. The results revealed that in addition to NTCP, the gene expression of multiple membrane proteins significantly enhanced HBV infection and that one protein, the one-way transmembrane glycoprotein neuropilin-1 (NRP1), could bind to HBV large surface proteins, thereby facilitating viral attachment to the cell surface. This NRP1–viral protein interaction enhances the ability of HBV to bind to NTCP, ultimately promoting viral infection (Yu et al., 2024).

**Figure 1:**
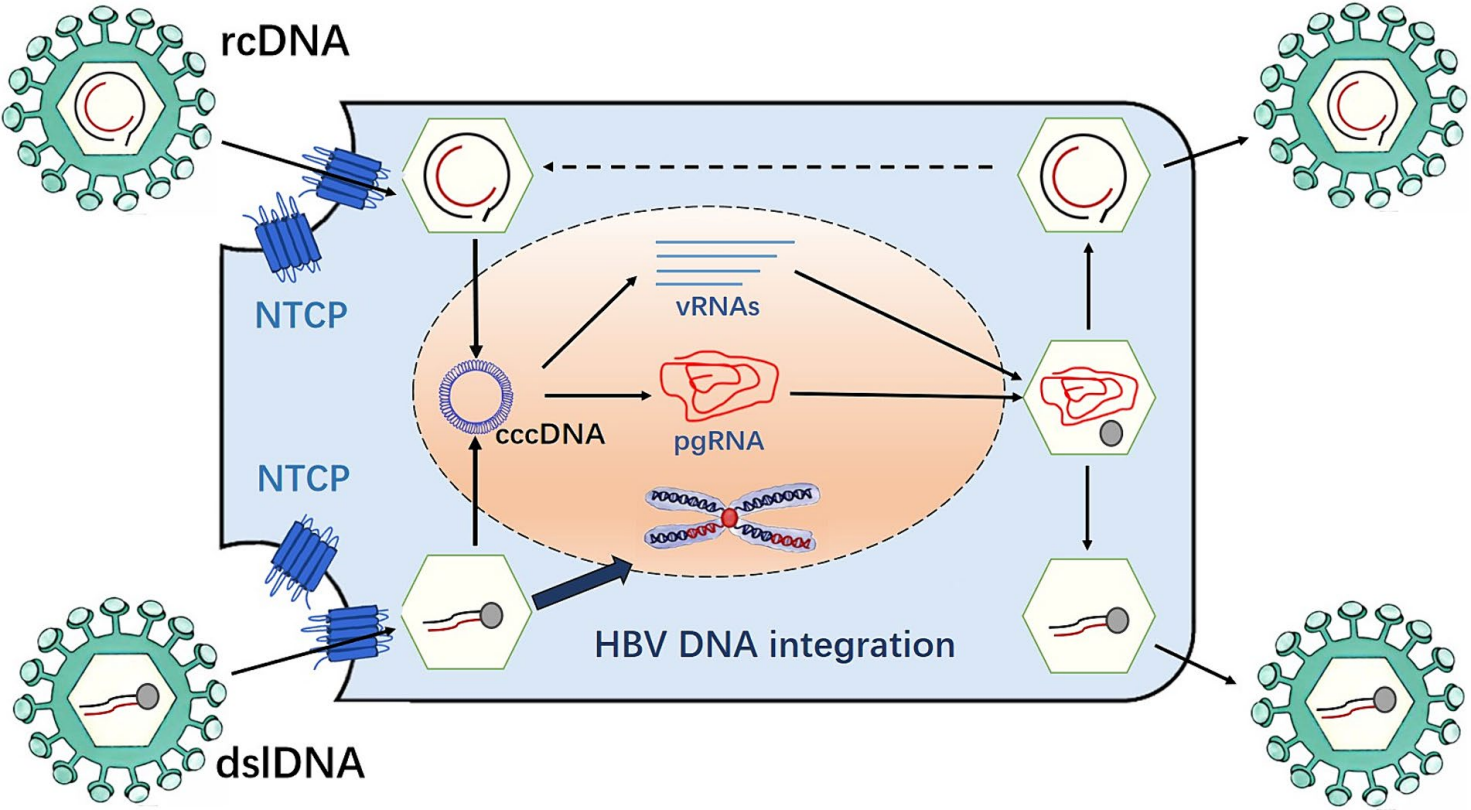
Replication of HBV genome.

Once the adhesion is successful, it means that HBV has entered the liver cells. The core part of HBV then enters the liver cells and removes its “nucleocapsid” (core antigen is HBcAG and E antigen is HbeAG) in the liver cytoplasm, exposing the core part, namely hepatitis B virus nucleic acid (HBV-DNA). The viral genome enters the nucleus through the nuclear pore, which is repaired by host factors and converted into the form of covalently closed circular DNA (cccDNA). This process involves multiple host proteins. The removal of the covalently attached HBV polymerase adduct is required to repair rcDNA (Guo et al., 2007; Luo et al., 2017), a process that involves multiple redundant factors. The HBV polymerase complex is initially removed by the redundant factors flap endonuclease 1 (FEN-1) and tyrosyl-DNA phosphodiesterase 2 (Wei and Ploss, 2020). The subsequent repair of negative and positive chains progresses independently. The repair of the negative strand by FEN-1 leads to the recognition and removal of the RNA primer, followed by the connection of the notch on the negative strand by DNA ligase 1 (LIG1) to complete the repair. The repair of the positive strand is similar to Okazaki fragment synthesis. First, the replication factor C complex recognizes the 3′ end of the positive chain and loads the proliferating cell nuclear antigen (PCNA) onto the prime-template junction. PCNA then recruits and activates POL delta using the PCNA interacting peptide sequence on POL delta, thereby filling the ssDNA gap and replacing the RNA primer at the 5′ end of the positive strand. Finally, FEN-1 recognizes and removes the replaced RNA primer and LIG1 connects to the gap left by the positive strand to complete the repair of the positive strand. This replication intermediate combines with host proteins to form the viral mini-chromosome, which serves as a template for viral RNA transcription. During this process, five viral mRNAs are produced, including pregenomic RNA (pgRNA), which is the main intermediate in HBV replication. The pgRNA is exported into the cytoplasm, and then inside the nucleocapsid, the pgRNA is reverse transcribed by the viral polymerase into new relaxed circular DNA (rcDNA). Alternatively, it can form double-stranded linear DNA (dslDNA) and plays an important role in integrating HBV DNA into the host genome. RcDNA can be delivered into the nucleus, increase the content of cccDNA, or bind to HBV surface proteins in the endoplasmic reticulum (ER), and be secreted outside the liver cells in the form of virions (Sidorkiewicz, 2001) (Figure 1). The cccDNA is responsible for controlling all the genetic information of HBV and instructing its replication. At this stage, HBV will use cccDNA as a template to transcribe all the information into mRNA. Subsequently, the genetic information on the mRNA is translated into various proteins such as HBV outer membrane protein (HBsAg), nucleocapsid protein (HBeAg, HBcAg), HBV-DNA polymerase, etc., followed by the shell formation. HBV is an unusual DNA virus because it resembles a retrovirus; therefore, it has a higher mutation rate than ordinary DNA viruses. Afterwards, HBV begins to assemble into complete particles in the cytoplasm, which are transported outside through the endoplasmic reticulum system. Some particles are returned to the nucleus to ensure the stable existence of cccDNA (Chemin and Zoulim, 2009).

## 4 Present status of HBV in the cells

HBV in cells has been observed in four states: relaxed circular structure rcDNA, the tight supercoiled structure cccDNA, virus integration state, and a possible extrachromosomal DNA (ecDNA) state (Chen et al., 2024). Each viral state has a different background of existence.

The viral genome in the relaxed state is most active during the early infection of hepatitis liver disease. The active replication relaxed state in cells is the main cause of viral hepatitis and can also lead to gradual changes in the organic structure of the liver. cccDNA is latent in the nucleus and acts as a supplementary library of rcDNA, which can produce pregenomic RNA (pgRNA) as a template for virus replication, thereby ensuring the stable existence of the virus in cells and effective reproduction. The HBV sequence in the integrated state is not necessary for virus replication. Therefore, the formation of this state may be closely related to the damage of the genome and the random insertion of the early viral genome. Once the HBV integration events occur, the instability of the genome increases. These factors work together with the external inflammatory environment of cells to select a group of cells with high adaptability and survival advantages, which lead to the occurrence and metastasis of cancer through multiple evolutions.

**Figure 2:**
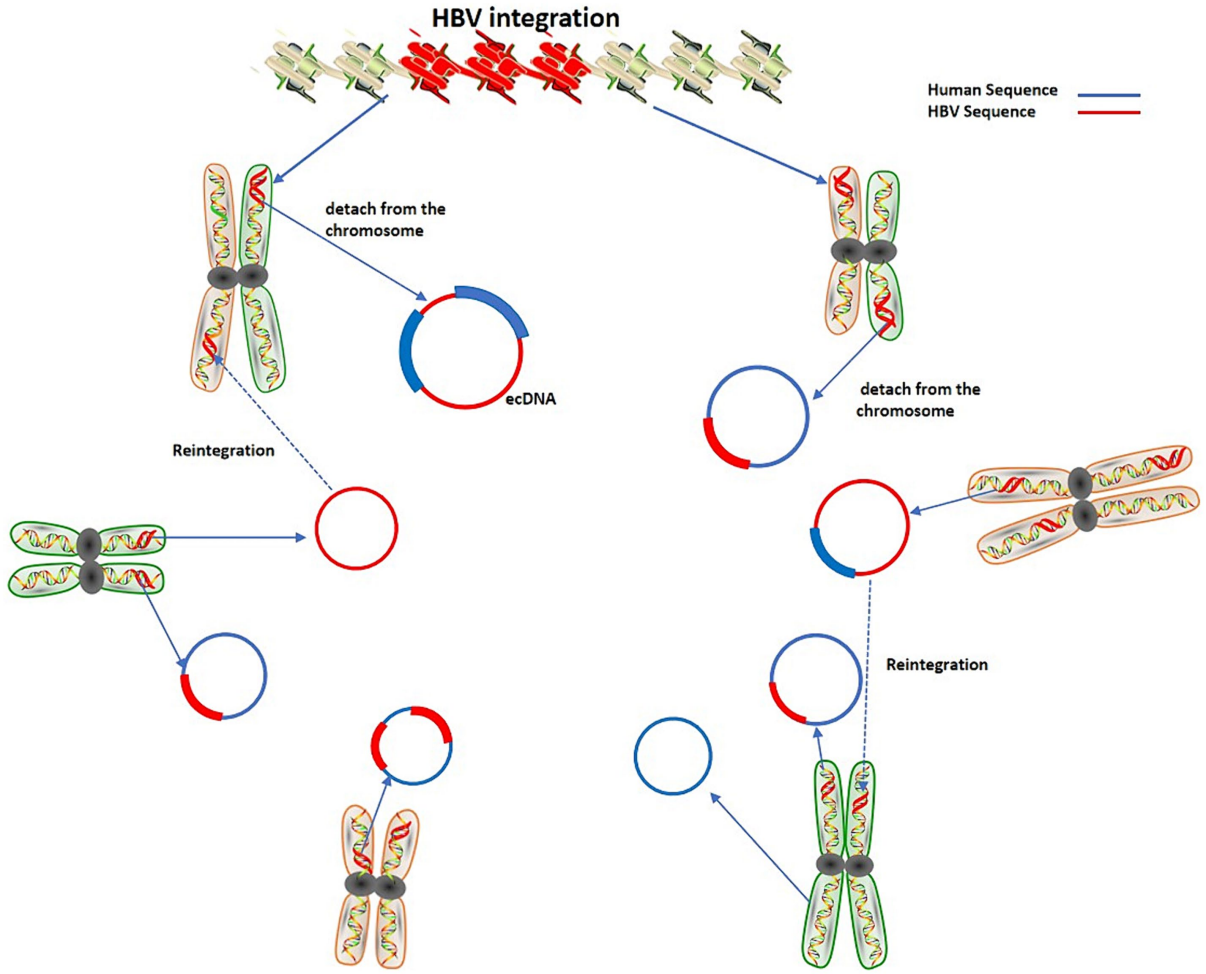
Dynamic process of HBV integration and extrachromosomal DNA.

cccDNA can produce rcDNA and double-stranded linear DNA (DSL-DNA). rcDNA can act as a virus genome library and can be transformed into cccDNA in the nucleus. It is known that virus integration occurs mainly due to DSL-DNA. Furthermore, the integrated virus DNA may detach from the chromosome and become ecDNA. This process may be dynamic that is, the detachment of DNA and its reintegration into the genome will continue to occur when the genome is unstable, thereby augmenting the instability level of the human genome (Ye et al., 2023) (Figure 2).

According to the existing data, the integration state of viruses into tumor cells is a crucial aspect of the virus genome. The research findings of Li et al. provide strong evidence for this speculation (Li et al., 2022). They found that HBV sequence with the integration site is about 49.5% of the total HBV DNA in the cells, indicating that the integrated form of HBV is important in HCC cells. Additionally, they reported that each cell contains about 3.2 copies of HBV and at least 1.6 copies of integrated HBV. The integration of HBV can lead to more frequent structural variations, including translocation (accounts for 56%) and deletions in the genome (Li et al., 2022). The fourth state is generally considered a part of the chromosome that has detached, and is often closely related to the overexpression of tumor genes, but the specific mechanism is yet to be fully explored (Hung et al., 2022). Therefore, the removal of cccDNA and the deletion of the virus integration sequence are crucial to the fundamental cure of hepatitis B. Although there are many antiviral drugs, it remains difficult to prevent the transformation of hepatitis to liver cancer. The fundamental reason is the lack of effective methods to eliminate viral integration and cccDNA.

## 5 Discovery of HBV integration events

Liver cancer is one of the most frequently occurring malignancies (Chen, 2015). HBV infection is known for its major contributions in causing liver cancer and HBV carriers have a higher risk of developing cancer than non-carriers (Ishikawa, 2010).

The integration of the HBV genome into the human genome in liver cancer was discovered as early as 1980 (Brechot et al., 1980). Researchers also found that the region around 1,800 bp of the cohesive end of the HBV genome is highly prone to integration events (Nagaya et al., 1987), and the HBV DNA deletion at the end is as high as 200 bp. HBV DNA integration occurs early in infection, and is already common in patients with hepatocellular carcinoma and cirrhosis with long-standing chronic hepatitis B (Brechot et al., 1980; Mason et al., 2010). A recent study reported that HBV DNA integration takes place before histologically observable liver damage in CHB patients (Mason et al., 2016). HBV DNA integration has been reported in children (as young as 5 months) who were congenitally infected with severe liver disease as well as in patients with acute HBV infection (Scotto et al., 1983; Yaginuma et al., 1987). These observations are corroborated with animal models of woodchucks and ducks, where integration was observed within days after infection (Summers et al., 2003). In fact, the first description of HBV DNA integration into the host cell genome was found in primary HCC tissues and HCC-derived cell lines, suggesting integrated HBV DNA as a causative factor (Koshy et al., 1981). Studies have investigated that HBV-related liver cancer is often accompanied by HBV virus integration events, and the positive rate of HBV virus integration detected in cancer tissue samples can be as high as 85–90% (Brechot et al., 2000). However, the positive rate of virus integration events in para-cancerous tissues is significantly lower than that in cancer tissue samples (Sung et al., 2012), thus the occurrence of liver cancer can be linked to viral integration.

## 6 Effect of HBV integration events

*In vitro* studies in a duck HBV infection model have shown that integration occurs preferentially at double-strand breakout sites (Bill and Summers, 2004). Integration of HBV genes in non-tumor tissues showed a dispersed distribution throughout the genome with only a few specific chromosomal hotspots or common relapse sites among patients, while tumor tissues showed some enrichment at specific genomic loci (Zhao et al., 2016). Recent studies using NGS have revealed that the structural arrangement of the integrated HBV DNA form affects the normal reading frame of the HBV genome, which in turn affects viral gene expression. Meanwhile, as the dslDNA form is only ~16 nt longer than the HBV genome length, integrated HBV DNA cannot produce pgRNA because of abnormal HBV genome, indicating that once viral integration occurs, it is difficult for the virus to replicate completely in cells. It has been investigated that Enhancer I (Enh I) is active in an integrated form, which leads to generation of HBx ORF (Shamay et al., 2001). Enh I, an enhancer identified in the HBV genome, has high activity in hepatogenic cell lines and can function together with heterologous promoters (Ben-Levy et al., 1989; Garcia et al., 1993; Honigwachs et al., 1989; Tang and McLachlan, 2001; Yu and Mertz, 2001; Zhang et al., 2024; Guo et al., 2023). It is essential for HBV transcription and viral promoter regulation (Honigwachs et al., 1989; Antonucci and Rutter, 1989; Zhou et al., 2016; Doitsh and Shaul, 1999).

In the integrated form, the 154 amino acids HBx may be truncated by at least three amino acids. However, studies have shown that HBx C-terminal truncated mutants (truncating 14 amino acids) are still effective in transcriptional transactivation (Kumar et al., 1996). Since the integrated form has no terminator at the 3′ end of the HBx ORF, it allows HBV to generate HBV-cell fusion transcripts. Integration is not necessary for viral replication, but it allows for a more stable presence of the viral genome. Long-term chronic inflammation can significantly increase the number of viral episomal DNA fragments in the host DNA during successive cell cycle death and proliferation, which can lead to the viral integration. As the viral integration can occur at different locations on different chromosomes, viral DNA fragments can affect the expression of targeted genes or even change the structure and function of proteins produced by genes, which in turn leads to the transformation of normal cells into malignant. The integration of viral DNA can also trigger carcinogenesis-related signaling cascades in cells through mutated viral proteins such as preS/S proteins or truncated X proteins, causing malignant cell transformation or transactivation (Zhang et al., 2021). In addition, the HBV genome has many enhancer structures that can activate gene expression through heterologous promoters (Qin et al., 2016). These enhancers can activate genes up to 100 Kb away from the site of cellular integration (Ozkal-Baydin, 2014). HBV integration can also induce recombination or deletion in the host chromosome near the integration site (Alvarez et al., 2021). Furthermore, viral integration can also lead to translocations, the production of fusion transcripts, and severe genome instability, hereby providing selective growth advantages during the growth of HBV-infected liver cells.

Previously, we found that 56% of virus integration was related to translocation and may replicate through the formation of a hairpin structure, resulting in inversion. These inversion events form a cross structure that is prone to fragmentation. Once the fragmentation occurs, it is easy to recombine with the broken region of the genome, causing variation in the translocation genome structure. These structural variations significantly increase the recombination frequency and hence lead to instability of the genome (Peneau et al., 2022; Jia et al., 2021). Furthermore, studies have shown that HBV-mediated rearrangements lead to frequent inter-chromosomal translocations and are often associated with a wide range of aberrations, including copy number changes in chr 4q (TERT), 5p, 6q, 8p, 16q, 9p (CDKN2A/B), 17p (TP53) and 13q (RB1), especially the ultra-early copy number gain on chr8q. Simultaneous HBV integration usually results in a complex structure, with each site exhibiting a varying end structure that may be mediated by different DNA repair mechanisms. Microhomologies (MHs) have been observed between the human genome and HBV genome near integration breakpoints, similar to the HPV integration pattern reported earlier (Hu et al., 2015). Facilitated by MHs flanking the breakpoint, HBV possibly hijacks the MH-mediated DNA repair pathways to fuse with the broken host genome and complete the integration process (Zhuo et al., 2021). Two repair mechanisms of MH-mediated end joining (MMEJ) have been found. MMEJ with pre-existing MH involves the deletion of one of the two repeats and all sequences between repeats (Yu and McVey, 2010). By contrast, SD-MMEJ uses a repeating primer located entirely on one side of the DSB to synthesize MH from scratch, and the sequence between the primer and the new MH template is copied and inserted into the break site (Yu and McVey, 2010).

In HCC, integration-induced copy number variations, such as ultra-early Chr8q amplification, may interact with the oncogenic effects of viral proteins and the overexpression of hotspot genes, leading to initial clonal expansion, shortening the progression time from normal cells to HCC, and accelerating the development of HCC (Qian et al., 2024).

## 7 Carcinogenesis of HBV integration event

It is believed that the mechanism of carcinogenesis of HBV-infected liver cells has the following three aspects: (1) viral DNA expression products, such as HBx protein, can induce proliferation and differentiation of liver cells; (2) chronic HBV infection caused by repeated stimulation of liver cell inflammation, which can lead to an inflammatory response and killing effect mediated by virus-specific T cells cause cumulative damage to the host hepatocyte genome and result in changes in genetic information (Hwang et al., 2020); (3) HBV DNA integrates into the hepatocyte genome, resulting in changes in the expression and function of endogenous target genes, as well as host chromosomal instability, which can lead to increased expression of certain cancer-related genes, such as: telomerase reverse transcriptase (TERT), mixed-lineage leukemia 4 (MLL4) and cyclin E1 (CCNE1) (Cui et al., 2023), or can also cause inactivation or decreased expression of tumor suppressor genes or changes in the expression of miRNAs. Despite inducing proliferation, differentiation and transformation of cells, HBV integration may directly regulate the transcription of adjacent cell genes, through cis-activation, causing cancer. Moreover, the integration of viral DNA can cause chromosomal recombination and loss of cellular DNA, eventually leading to malignant transformation and tumor formation in hepatocytes. It has been determined that virus integration can lead to the direct destruction of gene structure, promotion of abnormal transcriptional expression of genes, formation of fusion transcripts, and copying or deleting parts of the genome, which can cause genome instability, affect cell function and increases the chance of liver cancer.

## 8 Mechanisms of HBV integration

**Figure 3:**
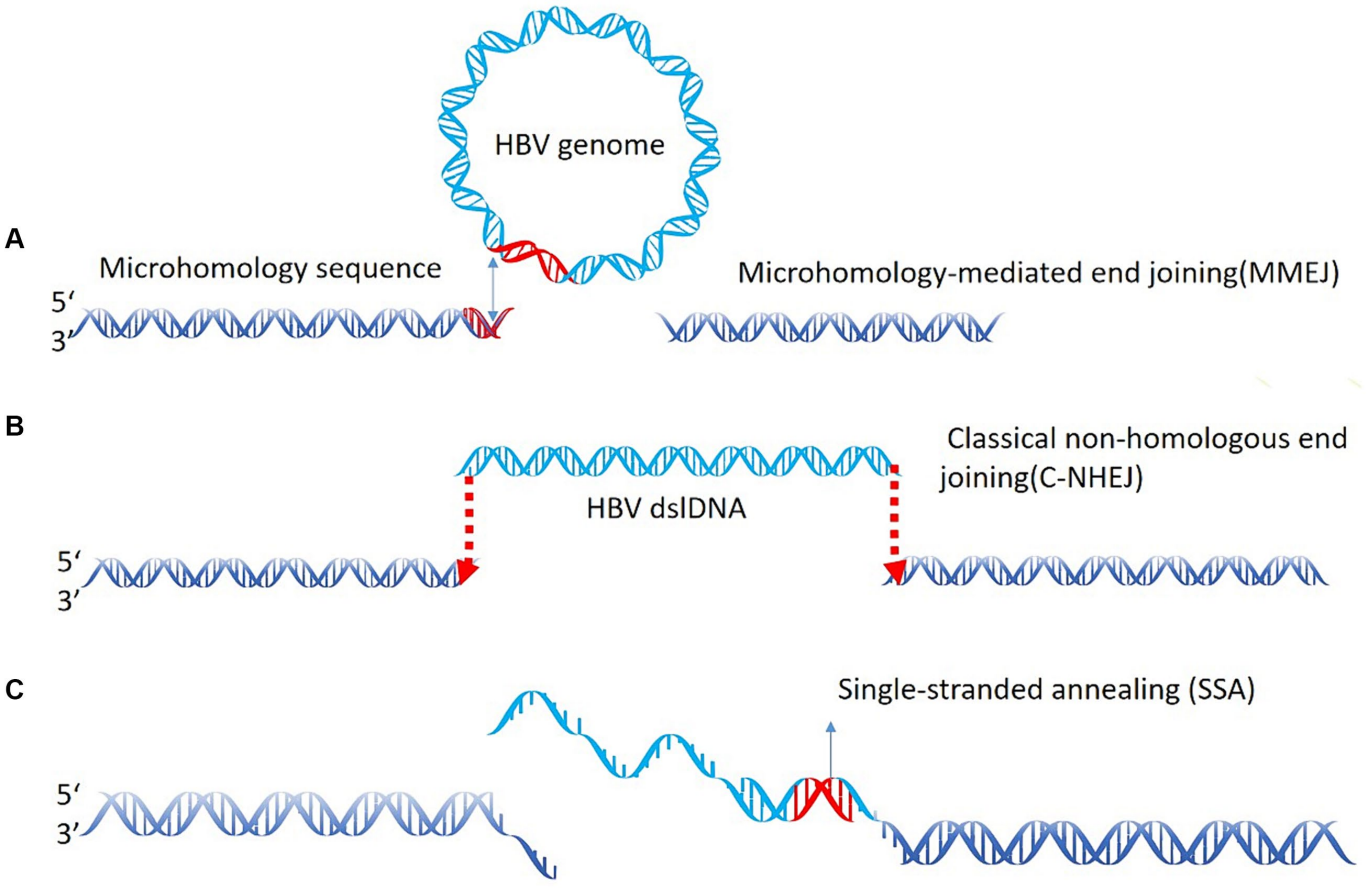
HBV integration mechanism. **(A)** Microhomology-mediated end joining (MMEJ) during virus integration joining; **(B)** classical non-homologous end joining (C-NHEJ); **(C)** the single-strand annealing mechanism.

In the field of virology and HCC, the virus integration mechanism has always been a focus of researchers. Studies have investigated that most HBV integration breakpoints occur within the range of 1,600–2,000 bp, but can also be present in other regions. These findings suggest that HBV integration into the host genome is not a simple process. During the replication process of HBV, in about 90% of the cases, the 18 nt RNA primer translocates to the DR2 sequence, leading to the synthesis of rcDNA. However, in the remaining 10% of the cases, the RNA primer binds to the DR1 region, synthesizing double-stranded linear DNA (dslDNA). HBV integration events generally occur in the host genomic DNA break region and are often accompanied by deletion. However, it remains unclear whether HBV DNA undergoes non-homologous end joining (NHEJ) or MMEJ during virus integration (Mladenov et al., 2016); however, recent studies utilizing next-generation sequencing (NGS) provide support for the latter (Zhao et al., 2016). Previously we found that HBV integration most likely occurs in dslDNA form through the classical NHEJ and MMEJ mechanisms (Sfeir and Symington, 2015; Lau et al., 2014; Budzinska et al., 2018). This study also revealed that there are breakpoints and integration events in the non-1800 bp, suggesting the integration of ssDNA into the human genome through the single-strand annealing mechanism (Figure 3). Moreover, integration of the closed circular HBV DNA into the human genome through the MMEJ mechanism has also been suggested (Hu et al., 2015; McVey and Lee, 2008).

## 9 Hotspots of virus integration

It has been investigated that the majority of integration events occur at or near common fragile sites. These sites of the genome are prone to deletions, breaks, chromosomal rearrangements, and gene amplification Furthermore, they also regulate the proliferation of genes, cell signaling, transduction, and cell viability. Additionally, these regions contain Alu sequences and microsatellite regions that are prone to development and increased genome instability (Feitelson and Lee, 2007). Sung et al. (2012) used the next-generation whole genome sequencing technology to examine the integration characteristics of HBV in 88 pairs of liver cancer samples. They revealed that the hotspot integration genes of HBV in liver cancer were TERT, KMT2B, and CCNE1. Furthermore, studies have found that majority of the high-frequency integrated oncogenes in tumor tissue are highly expressed, such as CTNND2, KMT2B, etc. While the high-frequency integrated tumor suppressor genes, such as PTPRD, UNC5D, etc., are relatively low-expressed when compared with oncogenes.

Research on HBV integration events has further confirmed that HBV integration events only contribute to the final malignant transformation of hepatocytes when they target host genes with oncogenic functions (Li et al., 2014). Likewise, when the HBV virus inserts the tumor suppressor gene MLL4 in the host genome, the MLL4 gene is activated, resulting in the malignant transformation of liver cells. Additionally, the integration of HBV DNA within the non-genic region of 8p11.21 can activate the Wnt signaling pathway by forming a new chimeric transcript, and this type of integration has been linked to poor clinical prognosis (Lau et al., 2014). Similarly, integration in the 8q24 region that is between the two oncogenes c-Myc and PVT1, occurs in 20% of patients with early-onset HCC (Yan et al., 2015). Furthermore, a large-scale study conducted on the integration rules and their clinical correlations in liver cancer reported that liver cancer patients with virus integration often have a worse prognosis (Li et al., 2013). In summary, the virus integration is a regular process and is closely linked to the occurrence and development of liver cancer. However, the rules of virus integration in different populations worldwide are still unclear, and the key pathways and mechanisms that promote tumor development are yet to be explored.

When the virus gets integrated into the intergenic region of the human genome, it leads to the instability of the genome near the integration region due to the change of the spatial structure of the genome sequence. Furthermore, integration can cause long-distance chromosome interaction and regulate long-distance related genes. It was found that mutations in the TERT promoter region can interact with the upstream 300 kb region long-distance (Min and Shay, 2016). Similarly, integration in the 8q24 region can remotely regulate MYC gene expression, thereby promoting tumorigenesis (Adey et al., 2013). Furthermore, integration of some viruses can directly interfere with the expression of related target genes through nascent chimeric transcripts, similar to LncRNA functions. Intergenic integrations possibly account for functional chimeric noncoding transcripts. The HBx-long interspersed nuclear elements 1 (LINE1) is another type of fusion transcript that acts as a noncoding RNA (Lau et al., 2014; Heikenwalder and Protzer, 2014). Lau et al. (2014) found that the function of HBx-LINE1 does not depend on the fusion protein and that it may affect the trans-activity of β-catenin. Subsequently, Liang et al. used a cell line model to confirm that HBx-LINE1 could act as a miR-122 sponge, which is a liver-specific miRNA and key regulator of liver disease (Liang et al., 2016).

These factors suggest the different integration modes may have varying effects on the genome level and the level of transcription and translation. Thus, their functions and mechanisms in the occurrence and development of liver cancer may be also different. Recently, we analyzed global virus integration hotspots and found that some hotspot genes have cross-ethnic characteristics. For example, the TERT gene and KMT2B gene are integration hotspot genes in Asian and European populations (Peneau et al., 2022; Cui et al., 2023). Therefore, it is suggested that virus integration is a common phenomenon that occurs in different people.

## 10 Pathway effects of HBV virus integration sites

Our research group discovered a series of new viral integration genes, including two new hotspot genes, expanding the knowledge of viral integration hotspots of HBV. From the perspective of virus integration, the event has a large random integration background, but also has some obvious hotspot genes such as TERT and KMT2B. Our research found that these hotspot genes are likely common in different races. Moreover, it provides an effective target for further research on the pathogenic mechanism and treatment of virus-integrated liver cancer (Cui et al., 2023). For a decade, it has been believed that virus integration would cause changes in genome structure, genome instability, abnormal expression of tumor suppressor genes and oncogenes, and abnormal expression of viral genes. It was found that virus integration can also be closely related to nerve axons and key synapses. Although the mechanism of viral integration affecting nerve axons and synaptic pathways still needs to be further studied, the veil of its close relationship with the nervous system has been gradually uncovered (Li et al., 2022).

## 11 HBV integration as a promising biomarker

The increasing data on HBV-DNA integration enhances our understanding of its cancer-promoting mechanism and highlights its potential as a detection marker. A recent meta-analysis identified 396 integration site-related genes in tumor samples, with 28 of these genes appearing in at least 10 patients (Lin et al., 2021). This meta-analysis highlights hotspots of HBV-DNA integration associated with the development of HCC. These genes may be developed into a panel for the diagnosis of liver cancer (Salpini et al., 2022).

Several studies have investigated HBV integration sites as useful non-invasive biomarkers for the early identification of HCC occurrence and recurrence (Li et al., 2019; Liu et al., 2021; Zhang et al., 2021). In 2019, our team demonstrated the presence of HBV integration site information in the blood samples of 20 HBV-infected patients, discovering a total of 87 different HBV integration sites. These sites were significantly enriched in tumor pathways. Although the source and proportion of the integrated fragments are not yet clear, this study shows that HBV DNA and integration sites in blood can be used as non-invasive viral integration detection biomarkers for liver cancer (Li et al., 2019). In 2021, another study applying a novel circulating single-molecule amplification and resequencing method confirmed that the majority of integration events detected in blood cell-free DNA originated from tumor tissue, further validating the potential of using HBV DNA integration as circulating tumor markers (Zheng et al., 2021). Regarding HCC recurrence, some studies have shown that viral integration was found in 10 cases (23.3%) of patients in the cell-free DNA after surgery, and 9 of these patients had liver cancer recurrence within 1 year. This indicates that viral integration in plasma cell-free DNA may develop into a new detection method for liver cancer (Li et al., 2020).

Furthermore, HBV DNA, HBV RNA, and cccDNA were also found in peripheral blood cells, and a large amount of HBV integration was identified in lymphocytes, including in cases of acute or latent HBV infection (Pontisso et al., 1984; Laure et al., 1985). Additionally, HBV viral integration was detected in peripheral blood mononuclear cell (PBMC) of patients with chronic hepatitis B using more sensitive methods (Laskus et al., 1999; Wang et al., 2008). In addition to PBMC, HBV integration was also found in hematopoietic stem cells (Shi et al., 2014).

**Figure 4:**
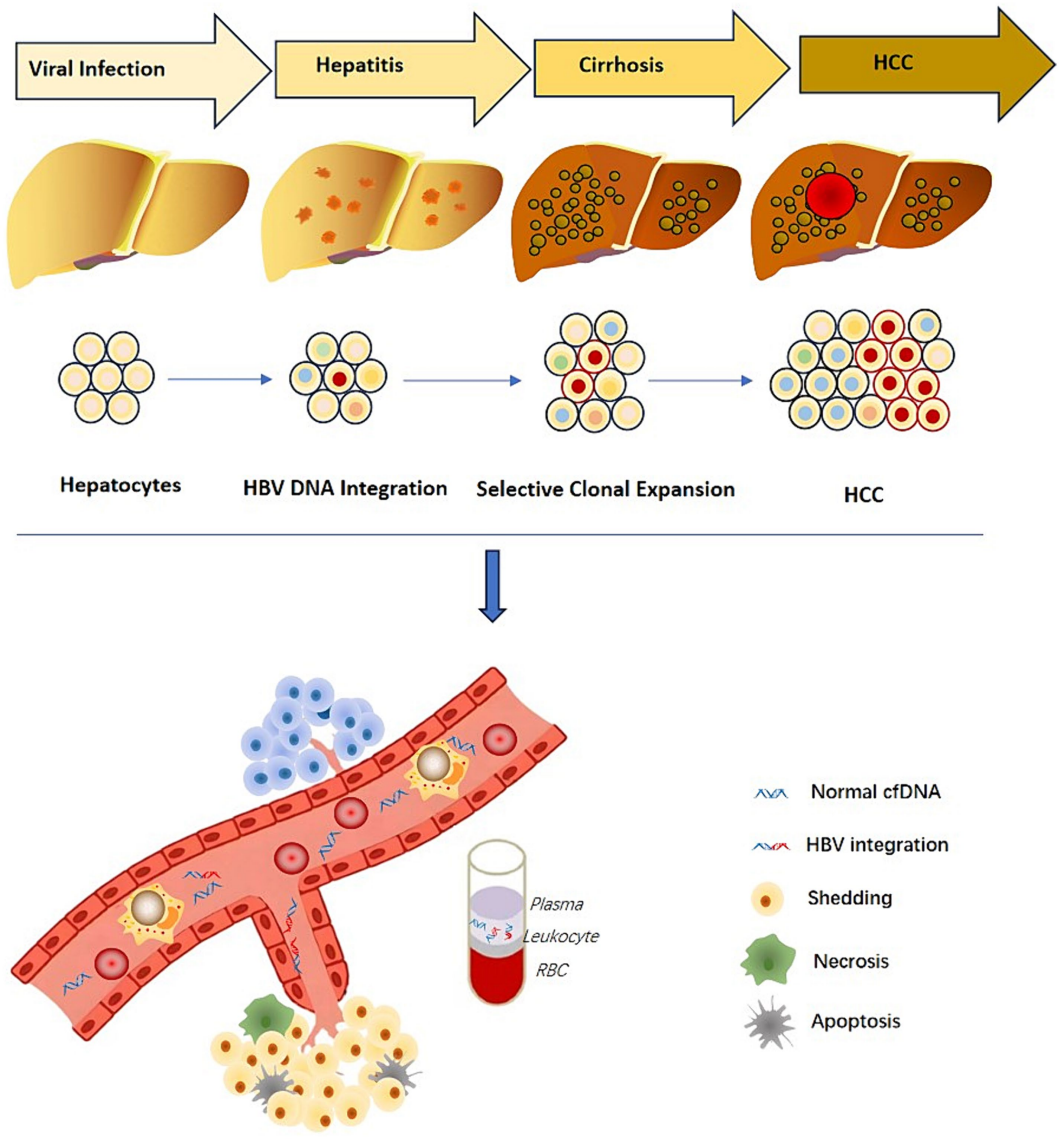
Cell free DNA associated with liver disease progression.

The discovery of HBV viral integration in plasma cell-free DNA and blood PBMC indicates that liver disease progression can be predicted through non-invasive detection. Thus, HBV integration information shows great promise as a biomarker (Figure 4).

## 12 The significance of third-generation sequencing and CRISPR/Cas9 technologies in explaining virus integration mode

**Figure 5:**
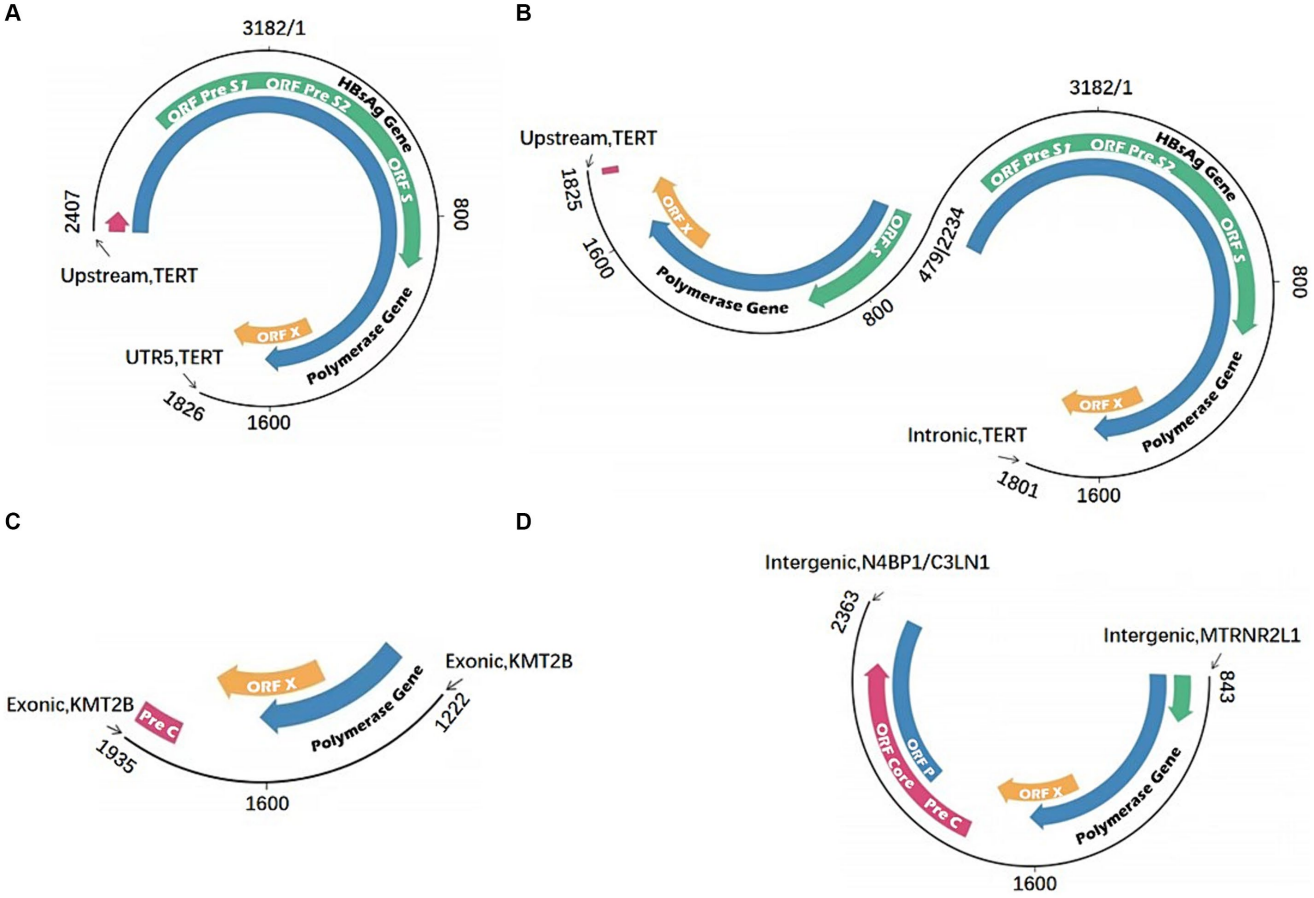
Multiple HBV integration mode. **(A)** HBV fragment inserted in the TERT gene; **(B)** HBV fragment with inversion structure inserted in the TERT gene; **(C)** HBV fragment inserted in the KMT2B gene; **(D)** HBV fragment inserted in the intergenic region.

The third-generation sequencing can read a length of 200 kb, making this technology efficient to span the virus integration fragments and determining the two end sites formed by the integration. These high throughput methods would obtain a complete and comprehensive virus integration mode. Various HBV virus integration patterns with high diversity and complexity have been found, including short-segment insertion and long-segment insertion in addition to inversion (Figure 5). It has significant advantages in the detection of complex regions of the genome, such as the highly complex HLA region, and can offer higher coverage and provide more accurate information (Westbrook et al., 2015; Mayor et al., 2015). Additionally, the full-length HCV genome can be directly sequenced through third-generation sequencing (Bull et al., 2016). Furthermore, investigating viral integration can sometimes be highly complex, as it is unknown whether these rearrangements occur prior to viral integration (integration of a defective HBV genome or HBV splice variants), after integration, or a combination of both. Li et al. carried out long-fragment virus integration analysis utilizing original HIVID technology, and have established a process based on third-generation sequencing (Li et al., 2022; Meng et al., 2019). Previous studies on virus integration were ineffective using longer viral genome fragments, making it difficult to analyze the chromatin accessibility and transcriptional expression abnormalities at the cell level caused by virus integration at specific sites. These limitations made it difficult to determine the carcinogenic pathways and mechanisms. However, the rapid progress in CRISPR/Cas9 technology has led to the development of targeted knock-in technology, enabling the knock-in of a 5.5 kb fragment into the genome (Yoshimi et al., 2016). The development of site-specific knock-in technology can rule out infection by external factors and more accurately study the impact of virus integration on genome, transcription and expression. Since virus integration has hotspots, it is more effective in determining the function of specific virus integration by performing a fixed-point integration design for specific hotspots.

## 13 Future research on HBV oncogenicity

The role of HBV integration in the initiation and progression of HCC is intricate. One of the most immediate consequences of viral integration is the alteration of genome structure. These changes in genome structure directly impact genome stability and result in modifications in chromatin accessibility. Different modes of virus integration can induce varying cellular functional changes. Therefore, elucidating the precise integration patterns of the virus and understanding the carcinogenicity of HBV integration are crucial steps in comprehending the overall process of virus integration-related carcinogenesis.

The development of third-generation technology and associated software has laid the groundwork for addressing this issue, and related research has progressively deepened. Once the virus’s integration patterns are clarified, research can advance to investigate the functional consequences of different integration modes. In this process, it will be important to establish cell lines and animal models with site integrations. By examining the diversity and regularity of virus integration modes, researchers can elucidate the specific impacts of key virus integration modes on cellular functions, encouraging more research efforts in this area. Additionally, understanding the mechanisms influencing viral integration modes will yield valuable insights.

Under the premise that the study of viral integration model is mature, researchers can use mathematical models to combine viral integration sites with internal viral integration models for the early diagnosis of liver cancer. Based on the demand of noninvasive diagnostic methods for clinical detection and continuous release of plasma-free DNA in the peripheral blood during liver cancer progression, combining the viral integration characteristics of plasma-free DNA with existing clinical data is of great significance for developing a noninvasive early detection model for liver cancer.

In the field of HBV-related liver cancer, the entry of HBV into the cell is the key mechanism. The blockade of HBV outside the cell at this stage can directly prevent the various effects of HBV. Therefore, additional studies on receptors used by HBV to enter cells are needed. When HBV enters the cell, cccDNA that performs multiple functions is formed, and it can be used as a template to form dslDNA. In the future, drug development and cccDNA detection may be particularly important for the treatment of HBV-related liver cancer. The integration of HBV can induce genomic instability, producing many virus particles to promote inflammation. These processes can be prevented only by removing the virus integration fragment. Therefore, the development of the CRISPR/CAS9 technique to shear integrated HBV sequence in the genome is of great therapeutic significance for liver cancer induced by viral integration.

As these mechanisms become clearer, corresponding therapeutic approaches can be developed. These may encompass gene editing methods, cell therapy strategies, and the creation of small molecule inhibitors, all with the potential to eliminate viral integration and inhibit its occurrence. As research in these areas progresses, continuous breakthroughs will emerge in early diagnosis, drug development, and mechanistic research in the field of liver cancer. Moreover, these advancements will extend to other virus integration-related fields, such as HPV integration-related cervical cancer and head and neck cancer. Various functional changes stemming from human immunodeficiency virus (HIV) integration will also continue to see progress, using the HBV research model as a foundation. Ultimately, these endeavors hold the promise of fundamentally addressing diseases related to viral integration and providing fresh insights and strategies to the broader field of viral treatment.
