# Erratum: The role of the hepatitis B virus genome and its integration in the hepatocellular carcinoma

**DOI:** 10.3389/fmicb.2024.1502267

**Published:** 2024-10-07

**Authors:** Frontiers Production Office

**Affiliations:** Frontiers Media SA, Lausanne, Switzerland

**Keywords:** hepatitis B virus integration, liver cancer, genomic instability, hepatitis B virus infection, hepatitis B virus

Due to a production error, Zhenzhen Guo, Sen Qiao, and Shulong Jiang were not listed as authors in the published article. The corrected **Author Contributions** statement appears below.

“WL: Conceptualization, Writing – original draft, Writing – review and editing. SW: Data curation, Writing – original draft. YJ: Investigation, Writing – original draft. XM: Investigation, Resources, Writing – original draft. ZG: Writing – original draft, Conceptualization, Investigation. SQ: Writing – original draft, Conceptualization, Investigation. SJ: Writing – original draft, Conceptualization, Investigation. QL: Conceptualization, Investigation, Writing – original draft. XC: Conceptualization, Writing – original draft.”

The publisher apologizes for this mistake. The original version of this article has been updated.

